# Association of end‐stage renal disease with HLA phenotypes and panel reactive antibodies in patients awaiting renal transplantation in Hunan Province

**DOI:** 10.1002/jcla.24251

**Published:** 2022-01-26

**Authors:** Lu Long, Qian Sun

**Affiliations:** ^1^ Department of Clinical Laboratory Xiangya Hospital Central South University Changsha China

**Keywords:** end‐stage renal disease, HLA phenotypes, panel reactive antibodies, polymorphism

## Abstract

**Objectives:**

To explore the immune‐related genetic susceptibility of human leukocyte antigen (HLA) alleles and their correlation with panel reactive antibody (PRA) generation during end‐stage renal disease (ESRD) progression.

**Materials and methods:**

Data of the expression patterns of HLA‐A, ‐B, and ‐DR alleles and PRAs of 347 ESRD patients awaiting renal transplantation in Hunan Province from 2015 to 2019 were retrospectively studied. The polymerase chain reaction with sequence‐specific primers was used for HLA genotyping and the enzyme‐linked immunosorbent assay for PRA detection. SPSS 21.0 software was used for all allele frequency and statistical analyses.

**Results:**

Thirteen HLA‐A, 25 HLA‐B, and 13 HLA‐DR alleles were expressed. The allele frequencies of HLA‐A2, ‐B48, ‐B52, and ‐B55 were significantly higher in the case group than in the control group (*p* < 0.05), whereas that of HLA‐B60 was significantly higher in the control group (*p* < 0.05). The frequency of HLA alleles in the PRA‐positive group was significantly higher in females than in males (*p* < 0.05). The allele frequencies of HLA‐A2, ‐B38, and ‐B46 were significantly higher in the PRA‐positive group than in the PRA‐negative one (*p* < 0.05), whereas that of HLA‐60 was significantly higher in the PRA‐negative group (*p* < 0.05).

**Conclusion:**

HLA‐A2, ‐B48, ‐B52, and ‐B55 may be the ESRD susceptibility alleles in Han Chinese patients in Hunan Province, whereas HLA‐B60 may be the protective allele. Patients carrying HLA‐A2, ‐B38, and ‐B46 are more likely to develop PRA positivity, whereas the opposite is true for those with HLA‐B60.

## Graphical abstract

Kidney transplantation using organs from relatives or donors is one of the main treatment methods for patients with end‐stage renal disease​(ESRD). The HLA genes, which are highly polymorphic, determine the compatibility of transplanted tissue with the recipient’s HLA phenotype. Panel reactive antibodies (PRAs) are anti‐HLA antibodies produced in the serum of patients, and the patient with positive PRA is sensitized and the risk of kidney transplantation failure will be high. HLA‐A2, ‐B48, ‐B52, and ‐B55 may be the ESRD susceptibility alleles in Han Chinese patients in Hunan Province, whereas HLA‐B60 may be the protective allele. Patients carrying HLA‐A2, ‐B38 and ‐B46 are more likely to develop PRA positivity, whereas the opposite is true for those with HLA‐B60.

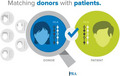

## 1 INTRODUCTION

Kidney transplantation using organs from relatives or donors is one of the main treatment methods for patients with end‐stage renal disease (ESRD).^1^ Human leukocyte antigens (HLAs), which are cell‐surface proteins encoded by the major histocompatibility complex in the human body, act mainly to regulate the immune response and immune regulatory functions. The HLA genes, which are highly polymorphic, determine the compatibility of transplanted tissue with the recipient's HLA phenotype, and their expression is closely related to the immune response.^2^ Panel reactive antibodies (PRAs) are anti‐HLA antibodies produced in the serum of patients. If the serum PRA of a kidney transplant recipient is greater than 10%, then that person is considered to be PRA positive, meaning that the patient is sensitized and the risk of kidney transplantation failure will be high.^3^ Because of the nonrandom expression patterns and linkage imbalance of HLA alleles, many studies have determined that the HLA phenotype of any individual has a certain correlation with that person's habitat region, race, disease occurrence, and PRA generation.^4^, ^5^, ^6^ It has also been shown that the long‐term survival rate of a kidney graft increases if the HLA types match to a higher degree.^7^

To determine the immune‐related genetic susceptibility of HLA alleles and their correlation with PRA generation during ESRD progression, in this study, we performed a retrospective statistical analysis using HLA phenotype and PRA data of 347 Han Chinese patients awaiting kidney transplantation in Hunan Province from 2015 to 2019. The results of this study will help toward ensuring that the generation of PRAs and the occurrence of transplant rejection are prevented for patients carrying susceptibility genes for ESRD, thereby improving the success rate of kidney transplantation.

## 2 MATERIAL AND METHODS

### 2.1 Patients

Patients with ESRD who were admitted to Xiangya Hospital of Central South University from 2015 to 2019 to await kidney transplantation in Hunan Province were selected for the study. The exclusion criteria were patients undergoing transfusion, those who had received transplants, and those with incomplete medical information. In total, the data of 347 patients were retrospectively analyzed as the case group. This group comprised 225 males and 122 females in the age range of 14–70 years (average age: 40.6 ± 11.3 years). On the basis of the PRA results, these 347 patients were further divided into a PRA‐negative group (*n* = 310 patients) and a PRA‐positive group (*n* = 37 patients). Additionally, 309 healthy individuals were randomly selected as the control group to determine the HLA allele distribution in a healthy population. This group comprised 157 males and 152 females in the age range of 13–69 years (average age: 43.4 ± 12.9 years). All study participants in the case and control groups were of Han Chinese nationality. This study was conducted under the criteria set forth in the Declaration of Helsinki and was approved by the Clinical Medical Ethics Committee of Xiangya Hospital, Central South University (Approval No. 202109005).

### 2.2 Reagents and instruments

The QIAamp DNA Blood Mini Kit was from QIAGEN Biotechnology Co., LTD.; the Micro SSP^TM^ HLA Typing Kit and Lambda Tray^TM^ HLA‐specific IgG Antibody Detection Kit were from One Lambda Biotechnology Co., LTD.; and the polymerase chain reaction (PCR) system was from Biometra Co., LTD.

### 2.3 DNA extraction

Approximately 3 ml of blood was collected intravenously from each participant into a vial containing EDTA as the anticoagulant. The QIAamp DNA Blood Mini Kit was then used to extract DNA from the peripheral blood lymphocytes. The DNA purity was determined to ensure that the A_260 nm_/A_280 nm_ was 1.6–1.8, and the DNA concentration was measured to be 40–100 ng/μl.

### 2.4 PCR amplification

The Biometra T1 Thermocycler was used for PCR amplification of the isolated DNA. The amplification cycles were set as follows: an initial 130 s at 97°C and 60 s at 63°C; then nine cycles of 10 s at 94°C and 60 s at 63°C; and finally 20 cycles of 10 s at 94°C, 50 s at 59°C, and 30 s at 72°C.

### 2.5 Electrophoresis of the PCR amplification products

The amplification products were separated on a 2% gel by electrophoresis at 150 V and 50 mA for 5 min. Thereafter, the bands were imaged using a UV analyzer, and One Lambda analysis software was used to determine the genotype.

### 2.6 ELISA screening for specific IgG antibody

The enzyme‐linked immunosorbent assay (ELISA) was performed with the HLA‐specific IgG Antibody Detection Kit according to the instructions of the manufacturer. The data were read using an ELISA tester, and the results were analyzed with One Lambda analysis software.

### 2.7 Statistical analysis

The HLA‐A, ‐B, and ‐DR allele frequencies were calculated directly. The Chi‐squared test was used to calculate the χ^2^ and *p* values, with a *p* value of less than 0.05 considered statistically significant. Relative risk (RR) values and odds ratios were calculated according to the Woolf formula. If the RR value is greater than 1, the allele is considered to be a risk factor for ESRD. If the RR value is less than 1, the allele is considered to play a protective role against ESRD development. If the RR value is equal to 1, the allele is not associated with ESRD. All statistical calculations were performed using SPSS 21.0 statistical software.

## 3 RESULTS

### 3.1 Comparison of HLA allele frequencies between the case and control groups

The HLA‐A, ‐B, and ‐DR genotypes were analyzed in 347 patients whose primary ailment was kidney disease that eventually progressed to ESRD.

#### 3.1.1 Allele frequency of the HLA‐A locus

In total, 13 HLA‐A alleles were detected in the case group, which had more HLA‐A29 and HLA‐A74 alleles (frequency: 0.14% and 0.29%, respectively) than the control group. By contrast, the control group had a higher frequency of the HLA‐A23 allele (0.32%). In both groups, the first three alleles were HLA‐A11, ‐A2, and ‐A24, which occurred at a frequency of 37.03%, 32.28%, and 17.58%, respectively, in the case group, and 39.32%, 36.21%, and 18.12%, respectively, in the control group. The frequency of HLA‐A2 in the case group was significantly higher than that in the control group (*p* < 0.05), whereas there was no statistically significant difference in the frequencies of the other alleles between the two groups (*p* > 0.05). The allele frequency results for this locus are presented in Table 1.

**TABLE 1:** Statistical analysis of allele frequencies of the HLA‐A locus

HLA‐A	case group (*n* = 347)		control group (*n* = 309)		χ^2^	*p*	RR	OR
	positive (*n*)	frequency (%)	positive (*n*)	frequency (%)				
A1	8	1.15	5	0.81	0.398	0.528	1.167	1.435
A2	224	32.28	162	26.21	9.924	0.002	1.274	1.653
A3	5	0.72	4	0.65	0.026	0.872	1.051	1.115
A11	257	37.03	243	39.32	1.889	0.169	0.891	0.776
A23	0	0.00	2	0.32	2.253	0.133	0.000	0.000
A24	122	17.58	112	18.12	0.084	0.772	0.978	0.954
A26	14	2.02	25	4.05	4.809	0.028	0.665	0.478
A34	1	0.14	1	0.16	0.007	0.935	0.945	0.890
A30	9	1.30	6	0.97	0.311	0.577	1.138	1.345
A31	11	1.59	18	2.91	2.727	0.099	0.708	0.529
A32	1	0.14	1	0.16	0.007	0.935	0.945	0.890
A33	39	5.62	39	6.31	0.298	0.585	0.938	0.877
A74	2	0.29	0	0.00	1.786	0.181	1.896	0.000
A29	1	0.14	0	0.00	1.126	0.380	0.665	0.000

#### 3.1.2 Allele frequency of the HLA‐B locus

In total, 25 HLA‐B alleles were detected in the case group, which had a higher frequency of HLA‐B76 (0.43%) than the control group. In both groups, the first three alleles detected were HLA‐B60, ‐B46, and ‐B13, occurring at the frequency of 20.17%, 17.72%, and 8.50%, respectively, in the case group, and 25.57%, 16.99%, and 10.68%, respectively, in the control group. The frequencies of the HLA‐B48, ‐B52, and ‐B55 alleles were significantly higher in the case group (*p* < 0.05), whereas that of the HLA‐B60 allele was significantly lower (*p* < 0.05). There were no statistically significant differences in the frequencies of the other alleles between the two groups (*p* > 0.05). The allele frequency results for this locus are presented in Table 2.

**TABLE 2:** Statistical analysis of allele frequencies of the HLA‐B locus

HLA‐B	case group (*n* = 347)		control group (*n* = 309)		χ^2^	*p*	RR	OR
	positive (*n*)	frequency (%)	positive (*n*)	frequency (%)				
B07	3	0.43	1	0.16	0.789	0.374	1.422	2.686
B08	5	0.72	4	0.65	0.026	0.872	1.051	1.115
B13	59	8.50	66	10.68	2.011	0.156	0.870	0.754
B18	1	0.14	2	0.32	0.463	0.496	0.629	0.444
B27	9	1.30	7	1.13	0.074	0.786	1.065	1.149
B35	12	1.73	15	2.43	0.807	0.369	0.834	0.702
B37	4	0.58	2	0.32	0.461	0.497	1.263	1.790
B38	24	3.46	11	1.78	3.646	0.056	1.318	2.013
B39	15	2.16	13	2.10	0.005	0.942	1.013	1.029
B44	2	0.29	7	1.13	3.446	0.063	0.417	0.250
B46	123	17.72	105	16.99	0.155	0.694	1.031	1.067
B48	19	2.74	6	0.97	5.568	0.018	1.462	2.925
B50	1	0.14	2	0.32	0.463	0.496	0.629	0.444
B51	39	5.62	38	6.15	0.177	0.674	0.952	0.903
B52	9	1.30	1	0.16	5.611	0.018	1.720	8.201
B54	16	2.31	13	2.10	0.063	0.802	1.045	1.101
B55	58	8.36	35	5.66	3.900	0.048	1.215	1.571
B57	3	0.43	2	0.32	0.102	0.749	1.135	1.339
B58	38	5.48	27	4.37	0.897	0.344	1.118	1.284
B60	140	20.17	158	25.57	7.672	0.006	0.813	0.646
B61	23	3.31	20	3.24	0.006	0.936	1.012	1.026
B62	38	5.48	38	6.15	0.289	0.591	0.939	0.877
B71	3	0.43	5	0.81	0.771	0.380	0.706	0.530
B75	47	6.77	40	6.47	0.051	0.821	1.025	1.054
B76	3	0.43	0	0.00	2.684	0.101	1.898	0.000

#### 3.1.3 Allele frequency of the HLA‐DR locus

In total, 13 HLA‐DR alleles were detected in the case group, in which the top three alleles HLA‐DR09, ‐DR04, and ‐DR12 were detected at a frequency of 17.29%, 15.56%, and 13.40%, respectively. Among the HLA‐DR alleles detected in the control group, the top three were HLA‐DR09, ‐DR15, and ‐DR04, occurring at a frequency of 20.87%, 13.27%, and 12.46%, respectively. However, there were no statistically significant differences in allele frequencies found between the two groups for the HLA‐DR locus. The allele frequency results for this locus are presented in Table 3.

**TABLE 3:** Statistical analysis of the allele frequencies of the HLA‐DR locus

HLA‐DR	case group (*n* = 347)		control group (*n* = 309)		χ^2^	*p*	RR	OR
	positive (*n*)	frequency (%)	positive (*n*)	frequency (%)				
DR01	2	0.29	4	0.65	0.930	0.335	0.628	0.442
DR04	108	15.56	77	12.46	3.108	0.078	1.150	1.362
DR07	10	1.44	14	2.27	1.261	0.261	0.781	0.625
DR08	57	8.21	57	9.22	0.465	0.496	0.934	0.869
DR09	120	17.29	129	20.87	3.564	0.059	0.864	0.738
DR10	7	1.01	4	0.65	0.518	0.472	1.207	1.570
DR11	62	8.93	42	6.80	2.239	0.135	1.155	1.383
DR12	93	13.40	72	11.65	1.064	0.302	1.090	1.205
DR13	22	3.17	23	3.72	0.311	0.577	0.919	0.842
DR14	66	9.51	62	10.03	0.114	0.736	0.969	0.936
DR15	85	12.25	82	13.27	0.359	0.549	0.950	0.898
DR16	34	4.90	24	3.88	0.837	0.360	1.120	1.290
DR17	28	4.03	28	4.53	0.206	0.650	0.940	0.881

### 3.2 Analysis of the PRA results in the case and control groups

#### 3.2.1 Comparison of the PRA positivity rate in patients of different genders

Among the 347 patients, 310 (89.34%) were negative and 37 (10.66%) were positive for PRAs. Among the 225 male patients, 216 (96.00%) were PRA negative and 9 (4.00%) were PRA positive. Among the 122 females, 94 (77.05%) were negative and 28 (22.95%) were positive for PRAs. The difference between males and females in terms of PRA generation was statistically significant (χ^2^ = 29.824, *p* = 0.000).

#### 3.2.2 Distribution frequency of the HLA‐A, ‐B, and ‐DR alleles in the PRA‐negative and ‐positive groups

The difference in HLA‐A2, ‐B38, ‐B46, and ‐B60 allele frequencies between the PRA‐negative and ‐positive groups was statistically significant when the count of genes less than 5 was excluded. In the PRA‐negative and ‐positive groups, the allele frequency of HLA‐A2 was 30.48% and 47.30%, respectively (χ^2^ = 16.335, *p* = 0.000); that of HLA‐B38 was 2.90% and 8.11%, respectively (χ^2^ = 5.564, *p* = 0.018); that of HLA‐B46 was 16.45% and 28.38%, respectively (χ^2^ = 8.219, *p* = 0.004); and that of HLA‐B60 was 21.13% and 12.16%, respectively (χ^2^ = 4.417, *p* = 0.036). The frequencies of these alleles are compared in Table 4.

**TABLE 4:** Distribution frequency of the HLA‐A, ‐B, and ‐DR alleles in different groups

Locus	PRA‐negative group (*n* = 310)		PRA‐positive group (*n* = 37)		χ^2^	*p*	RR
	n	frequency (%)	*n*	frequency (%)			
A2	189	30.48	35	47.30	16.335	0.000	9.609
B38	18	2.90	6	8.11	5.564	0.018	2.605
B46	102	16.45	21	28.38	8.219	0.004	2.390
B60	131	21.13	9	12.16	4.417	0.036	0.475

## 4 DISCUSSION

As a very important genetic marker of human beings, the HLA system comprises a group of genes that determine whether the transplanted tissue is compatible with the recipient's HLA phenotype and is closely related to the immune response.^8^ For patients who progress from primary nephropathy to ESRD and require kidney transplantation, the degree of match or mismatch of the HLA types will directly affect the success rate of organ transplantation and subsequent immunosuppressive therapy.^9^ According to “China's Common and Well‐documented Alleles, and Confirmed HLA Allele Table (CWD) version 2.3,” 165 HLA antigens, including those encoded on 28 HLA‐A loci, 62 HLA‐B loci, and 24 HLA‐DR loci, have been detected in the country to date. The high level of genetic polymorphism, co‐dominant gene expression, and shortage of donors pose a serious challenge to clinical kidney transplantation in China.^10^ In this retrospective study, a total of 14 (14/28) HLA‐A alleles, 25 (25/62) HLA‐B alleles, and 13 (13/24) HLA‐DR alleles were detected in the case and control groups of Han Chinese in Hunan Province. In the case group, the top three alleles with the highest frequencies in each of the three loci were HLA‐A2 (32.28%), ‐A11 (37.03%), and ‐A24 (17.58%); HLA‐B60 (20.17%), ‐B46 (17.72%), and ‐B13 (8.5%); and HLA‐DR09 (17.29%), ‐DR04 (15.56%), and ‐DR12 (13.40%), respectively. However, the distribution of HLA genotypes in this study was quite different from that in other areas in China, where the top three alleles with the highest frequency in the three loci were found to be HLA‐A11 (23.83%), ‐A24 (17.16%), and ‐A2 (11.36%); HLA‐B40 (14.08%), ‐B46 (12.20%), and ‐B58 (8.50%); and HLA‐DR9 (17.52%), ‐DR12 (10.57%), and ‐DR15 (9.70%), respectively.^11^

Many studies have shown that the immune‐related genetic susceptibility of the HLA system is associated with various diseases, and many HLA genes have also been reported to be associated with the RR of primary kidney disease.^12^, ^13^ Because of the random expression patterns and linkage imbalance of the HLA alleles, their genetic susceptibility differs in different geographic regions and nationalities.^14^

In this study, the allele frequencies of HLA‐A2, ‐B48, ‐B52, and ‐B55 were significantly higher in the case group than in the control group (*p* < 0.05), whereas that of HLA‐B60 was significantly higher in the control group (*p* < 0.05), indicating that HLA‐A2, ‐B48, ‐B52, and ‐B55 may be independent susceptibility alleles for ESRD in patients with primary nephropathy in Hunan Province. By contrast, HLA‐B60 may be a protective allele. In an analysis of protective and susceptibility HLA alleles and haplotypes in a single‐center study of patients with ESRD in Jiangsu Province (China), Pan et al. found that HLA‐A11, ‐A31, ‐B15, ‐B55, ‐B39, ‐DR11, and ‐DR12 emerged as susceptibility alleles, whereas HLA‐DR15 was protective. The haplotype A11‐B15‐DR11 containing three susceptibility alleles was regarded as the most susceptible one.^15^ Doxiadis et al. reported that the frequencies of the HLA‐B35 and ‐DR5 antigens were significantly increased, whereas those of the HLA‐B7, ‐B8, ‐DR2, and ‐DR3 antigens were significantly decreased, in 1620 Caucasian European patients with ESRD due to primary IgA nephropathy. In those patients, HLA‐B35 and ‐DR5 may be the susceptibility alleles for ESRD.^16^ In a study of patients in Kuwait, Mosaad et al. found that HLA‐B8 may be a susceptibility allele, and HLA‐A28 and ‐DR11 were the protective alleles, during the occurrence and development of ESRD.^17^ The differences in frequency distribution and genetic susceptibility data between this study and other studies in China and other regions and countries may be caused by differences in the distribution of HLA alleles in different geographic regions, in the research cases, and so on.

In the PRA‐positive group of this study, the HLA allele frequency in the females was 22.95%, which was significantly higher than that in the males (4%; *p* < 0.05), indicating that there were statistically significant differences in PRA production between the different gender groups. The reason that the female patients were more likely to develop PRAs may be due to an increased exposure to foreign antigen stimulation during pregnancy, and this difference is consistent with that described in other studies.^18^, ^19^ Moreover, the allele frequencies of HLA‐A2, ‐B38, and ‐B46 were significantly higher in the PRA‐positive group than in the PRA‐negative group (*p* < 0.05), whereas that of HLA‐B60 was significantly higher in the PRA‐negative group (*p* < 0.05), suggesting that different alleles may have different rates of developing PRA. Although HLA‐B60 and ‐B46 were the first two alleles detected in the HLA‐B locus in this study, their frequencies differed in the PRA‐positive and ‐negative groups. This allele frequency difference was also found in another local study.^20^ The association of HLA‐B60 with a significantly reduced risk of sensitization has been reported.^21^ The inference about the correlation between HLA alleles and PRA generation in this study—that is, patients with HLA‐A2, ‐B38, and ‐B46 alleles may be more likely to develop PRA positivity, whereas those with HLA‐B60 may be more likely to develop PRA negativity—may be used to predict the possibility of ESRD and PRA generation in patients with primary kidney disease. For PRA‐positive patients, donors with protective alleles should be preferentially selected to reduce the risks of immune‐based transplant rejection and PRA generation. Therefore, the inferences made in this study can provide more effective guidance in the selection of donors for clinical kidney transplantation and help to improve the success rate of this treatment process.

## 5 CONCLUSION

In conclusion, HLA‐A2, ‐B48, ‐B52, and ‐B55 may be independent susceptibility alleles for ESRD in patients with primary nephropathy in Hunan Province, whereas HLA‐B60 may be a protective allele. The generation of PRAs correlates with the patient gender and HLA allele distribution, with female patients being more likely to develop such antibodies. Patients with the HLA‐A2, ‐B38, and ‐B46 alleles may be more likely to develop PRA positivity, whereas those carrying the HLA‐B60 allele are more prone to be PRA negative. The finding of HLA‐A2 as an independent susceptibility allele for ESRD and a risk factor for developing PRA positivity indicates that patients carrying this allele may have a poor prognosis. By contrast, patients carrying the protective HLA‐B60 allele against ESRD development would not generate PRAs easily and thus may have a better prognosis.

## AUTHOR CONTRIBUTIONS

QS contributed to study conceptualization, sample processing, interpretation of results, and writing—first draft of manuscript and final manuscript approval. LL: statistical analysis, study design, and interpretation of results. Both authors have reviewed and approved the final version of the manuscript.

## Data Availability

Data sharing was not applicable to this article, as no datasets were generated or analyzed during the current study.
